# High-Sensitivity, Low Detection Limit, and Fast Ammonia Detection of Ag-NiFe_2_O_4_ Nanocomposite and DFT Study

**DOI:** 10.3390/nano15141088

**Published:** 2025-07-14

**Authors:** Xianfeng Hao, Yuehang Sun, Zongwei Liu, Gongao Jiao, Dongzhi Zhang

**Affiliations:** College of Control Science and Engineering, China University of Petroleum (East China), Qingdao 266580, China; haoxf@upc.edu.cn (X.H.);

**Keywords:** Ag-NiFe_2_O_4_, NH_3_ sensor, low detection limit, DFT, simulations

## Abstract

Ammonia (NH_3_) is one of the characteristic gases used to detect food spoilage. In this study, the 10 wt% Ag-NiFe_2_O_4_ nanocomposite was synthesized via the hydrothermal method. Characterization results from SEM, XRD, and XPS analyzed the microstructure, elemental composition, and crystal lattice features of the composite, confirming its successful fabrication. Under the optimal working temperature of 280 °C, the composite exhibited excellent gas-sensing properties towards NH_3_. The 10 wt% Ag-NiFe_2_O_4_ sensor demonstrates rapid response and recovery, as well as high sensitivity, towards 30 ppm NH_3_, with response and recovery times of merely 3 s and 9 s, respectively, and a response value of 4.59. The detection limit is as low as 0.1 ppm, meeting the standards for food safety detection. Additionally, the sensor exhibits good short-term repeatability and long-term stability. Additionally, density functional theory (DFT) simulations were conducted to investigate the gas-sensing advantages of the Ag-NiFe_2_O_4_ composite by analyzing the electron density and density of states, thereby providing theoretical guidance for experimental testing. This study facilitates the rapid detection of food spoilage and promotes the development of portable food safety detection devices.

## 1. Introduction

NH_3_ is a colorless gas with a pungent odor, widely present in nature and industrial production [1]. In the field of fish storage and preservation, the generation and accumulation of ammonia are directly related to the spoilage process of fish, as ammonia is one of the final products of protein decomposition [2,3,4]. Changes in ammonia concentration are commonly used as a key indicator to assess the freshness of fish. After fish die, the proteins and other nitrogen-containing compounds in their bodies begin to decompose [5]. This process is primarily driven by microorganisms such as bacteria and fungi, which break down the proteins in the fish tissue into amino acids, and further decompose the amino acids into ammonia, hydrogen sulfide, and other volatile compounds. Therefore, timely and accurate detection of ammonia produced during the spoilage process of fish provides data support for accurately assessing fish freshness and optimizing storage conditions.

Commonly used toxin detection sensors include optical sensors [6], colorimetric sensors [7], electrochemical sensors [8], and semiconductor sensors [9]. Among these, metal oxide semiconductor gas sensors have garnered extensive attention due to their low cost, high sensitivity, and rapid response and recovery characteristics [10]. NiFe_2_O_4_ is a spinel-structured composite oxide and is one of the research hotspots in the field of gas-sensing due to its high sensitivity, low cost, and good thermal stability [11,12,13,14,15,16]. Rezaeipour et al. [17] prepared porous nickel ferrite nanoparticles using a hydrothermal method. The sensor employs a side-heating structure, with a glass plate adhered to the heating element to fabricate a low-cost sensing component. This configuration allows for the evaluation of the sensor’s acetone-sensing characteristics at various operating temperatures. The selectivity of the sensor was tested towards multiple gases, including methanol, xylene, dichloromethane, and toluene. At an operating temperature of 190 °C, the sensor exhibited a high response value of 78% towards 100 ppm acetone and a response value of 67% towards ethanol. The sensor exhibits satisfactory stability and repeatability, and it can swiftly respond to acetone within 30 s. In a related study, Zhang et al. [18] fabricated NiFe bimetallic organic framework (MOF) octahedra using a straightforward reflux technique and then transformed them into NiFe_2_O_4_ nanooctahedra through calcination. The synergistic presence of phthalic acid (PTA) and 3,3-diaminobiphenyl (DAB) promoted the creation of NiFe bimetallic MOF octahedra. Following a thermal treatment, the NiFe_2_O_4_ nanoparticles (30 nm) experienced an approximate 85% weight loss, evolving into porous nano-octahedra with a hollow interior. Utilizing these NiFe_2_O_4_ nano-octahedra, a sensor was developed that exhibited remarkable toluene gas-sensing capabilities, characterized by notable long-term stability over 30 days, swift response, and brief recovery times (25 s/40 s), along with a low detection threshold of 1 ppm at 260 °C. Among all the strategies, noble metal doping is undoubtedly a highly appealing approach, which can be attributed to the chemical and electronic sensitization behaviors of noble metals [19]. Owing to their unique catalytic mechanisms, noble metals can significantly reduce the adsorption activation energy on the surface of the doped materials, thereby enhancing the sensitivity, response, and selectivity of the sensors [20].

In this investigation, Ag-NiFe_2_O_4_ nanocomposites were synthesized employing a hydrothermal synthesis approach. Various characterization techniques, including SEM, XPS, and XRD, were employed to analyze the composites, confirming the successful synthesis of Ag-NiFe_2_O_4_. The gas-sensing capabilities of the sensors were evaluated, revealing that the sensor doped with 10 wt% Ag-NiFe_2_O_4_ exhibited the highest response at 280 °C. Additionally, corroborated by DFT calculations, simulations authenticated the enhanced ammonia adsorption mechanism of the Ag-NiFe_2_O_4_ composite.

## 2. Experimental

### 2.1. Materials

The anhydrous ethanol (C_2_H_6_O, 99.7%) and polyvinylpyrrolidone (PVP, average molecular weight of 1,300,000) used were from Maclin Biochemical Co., Ltd. (Shanghai, China). The producer of silver nitrate (Ag(NO_3_)_2_, 99.8%) is Sinopharm Chemical Reagent Co., Ltd. (Shanghai, China). Nickel nitrate hexahydrate (Ni(NO_3_)_2_·6H_2_O, 98%), ferric nitrate nonahydrate (Fe(NO_3_)_3_·9H_2_O, 98.5%), sodium hydroxide (NaOH, 99%), and sodium borohydride (NaBH_4_, 99%) were from Shanghai Hushi Laboratory Equipment Co., Ltd. (Shanghai, China). Sodium citrate (C_6_H_5_O_7_Na_3_, 98%) is produced by Shanghai Aladdin Biochemical Technology Co., Ltd. (Shanghai, China).

### 2.2. Synthesis of Ag-NiFe_2_O_4_ Nanocomposites

Add 0.1308 g of Ni(NO_3_)_2_·6H_2_O and 0.3636 g of Fe(NO_3_)_3_·9H_2_O to 40 mL of deionized water while stirring continuously. Stir on a magnetic stirrer until a transparent solution is formed, then gradually add 1 mol/L NaOH solution dropwise until the pH of the solution reaches 12. Stir for 3 h. After stirring, transfer the mixture into a hydrothermal reactor and heated at 180 °C for 18 h. Subsequently, collect the precipitate and wash it multiple times to remove impurities, then dry it in an oven at 60 °C for 24 h to obtain NiFe_2_O_4_ nanoparticles.

Add 1 mL of 10 mol/L Ag(NO_3_)_2_ solution to a beaker, followed by the addition of 1 mL of 1 mol/L PVP. Agitate the mixture within an ice-water bath for a duration of 1 h, then add 1.5 mL of 10 mol/L C_6_H_5_NaO_7_ solution and stir for another hour. While stirring in the ice-water bath, gradually add 0.16 mL of 0.1 mol/L NaBH_4_ solution dropwise. After the reaction is complete, collect the precipitate and wash it multiple times to remove impurities, then dry it to obtain silver nanoparticles.

In the synthesis of Ag-NiFe_2_O_4_ composites, 0.3 g of NiFe_2_O_4_ particulate matter was dispersed in 10 mL of anhydrous ethanol. Based on the mass of NiFe_2_O_4_ and mass ratios of 5 wt%, 10 wt%, and 15 wt%, 0.015 g, 0.030 g, and 0.045 g of silver nanoparticles were added to the three respective NiFe_2_O_4_ suspensions. After ultrasonic treatment, the mixtures were stirred at 60 °C for 12 h. The synthesized composites with varying silver nanoparticle loadings were, respectively, defined as 5 wt% Ag-NiFe_2_O_4_, 10 wt% Ag-NiFe_2_O_4_, and 15 wt% Ag-NiFe_2_O_4_.

## 3. Results and Discussion

### 3.1. Characterization of the Prepared Material

**Figure 1:**
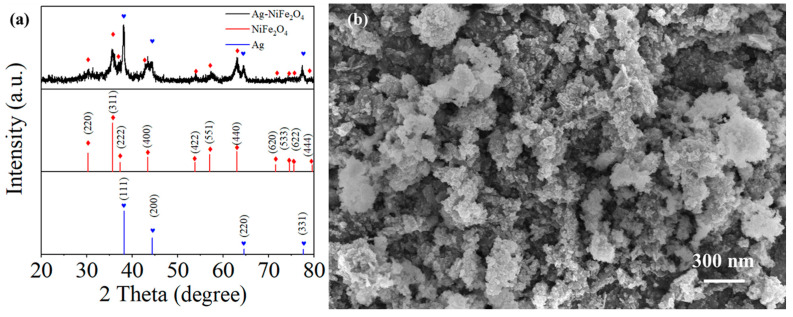
(**a**) XRD and (**b**) SEM of 10 wt% Ag-NiFe_2_O_4_-samples.

The XRD pattern of Ag-NiFe_2_O_4_ is shown in Figure 1a. The diffraction peaks of NiFe_2_O_4_ are concentrated at 2θ = 30.294°, 35.685°, 37.329°, 43.373°, 53.819°, 57.375°, 63.013°, 71.504°, 74.572°, 75.584°, and 79.591°, corresponding to the (2 2 0), (3 1 1), (2 2 2), (4 0 0), (4 2 2), (5 1 1), (4 4 0), (6 2 0), (5 3 3), (6 2 2), and (4 4 4) crystal planes (JCPDS 74-2081). The diffraction peaks of Ag are located at 2θ = 38.200°, 44.400°, 64.600°, 77.597°, and 81.755°, corresponding to the (1 1 1), (2 0 0), (2 2 0), (3 1 1), and (2 2 2) crystal planes (JCPDS 87-0720). This indicates the successful preparation of Ag-NiFe_2_O_4_. The SEM image of Ag-NiFe_2_O_4_ is shown in Figure 1b. Ag-NiFe_2_O_4_ appears in the form of nanoparticles. The average particle size of the nanoparticles is 100–200 nm. The material exhibits good crystallinity and a uniform nanostructure. The larger specific surface area provides a foundation for the preparation of high-performance gas sensors.

**Figure 2:**
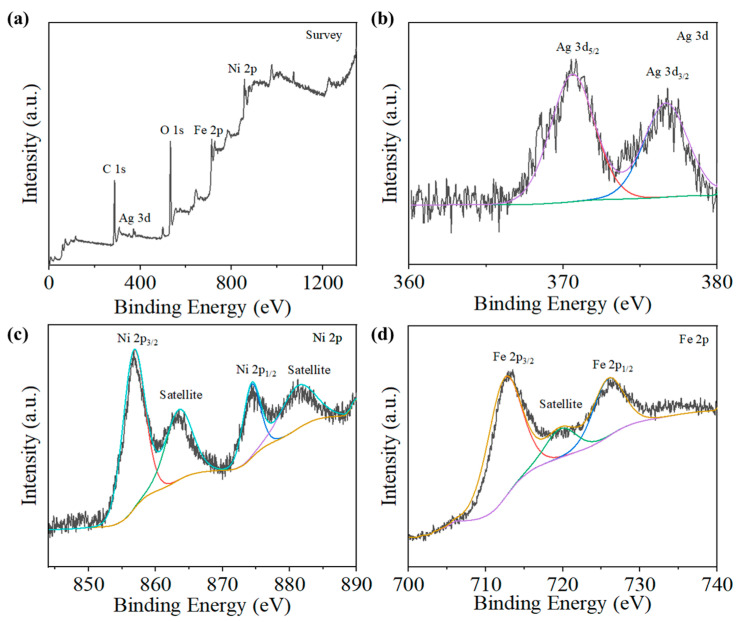
The XPS plot of Ag-NiFe_2_O_4_ sample: (**a**) full spectrum scanning, (**b**) Ag 3d, (**c**) Ni 2p, (**d**) Fe 2p.

The elemental composition of the 10 wt% Ag-NiFe_2_O_4_ nanocomposite was characterized using X-ray photoelectron spectroscopy (XPS, Thermo Fisher Scientific, Waltham, MA, USA). The XPS analysis curve of the 10 wt% Ag-NiFe_2_O_4_ sample is shown in Figure 2. As illustrated in Figure 2a, the full spectrum of the 10 wt% Ag-NiFe_2_O_4_ sample confirms the presence of Ag, Ni, and Fe elements in the composite. In the Ag 3d spectrum, the two peaks at 370.63 eV and 376.68 eV correspond to the Ag 3d_5/2_ and Ag 3d_3/2_ orbitals, indicating the presence of metallic Ag (Figure 2b). In the Ni 2p spectrum, the characteristic peaks at 856.75 eV and 847.33 eV correspond to Ni 2p_3/2_ and Ni 2p_1/2_ orbitals, respectively (Figure 2c). In addition to the main peaks, satellite peaks were also observed. These satellite peaks, which typically appear on the higher binding energy side of the main peaks, are characteristic of Ni^2+^. Their presence further confirms that the oxidation state of Ni is +2. As shown in Figure 2d, the Fe 2p spectrum displays two main peaks, with characteristic peaks at 712.42 eV and 725.88 eV corresponding to Fe 2p_3/2_ and Fe 2p_1/2_ orbitals, respectively. Satellite peaks were also observed, confirming that the oxidation state of Fe is +3.

### 3.2. NH_3_ Sensitive Properties

**Figure 3:**
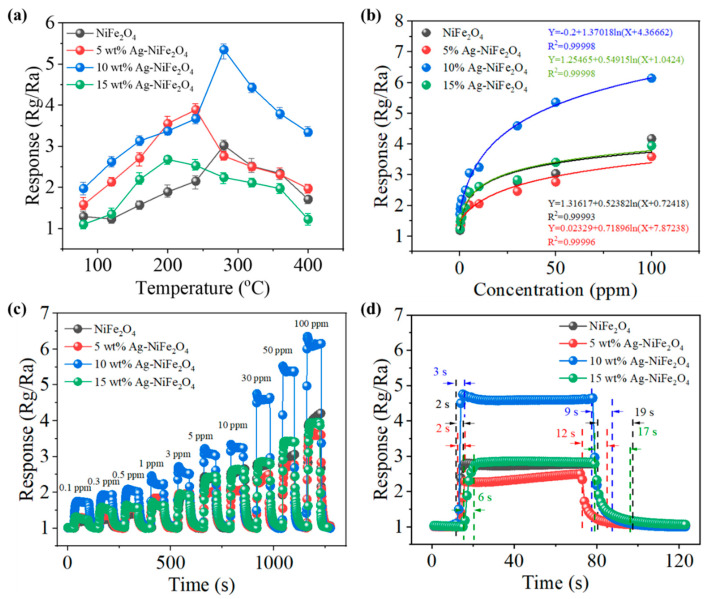
(**a**) Response values of Ag-NiFe_2_O_4_ sensor with different mass ratios to 50 ppm NH_3_ at different temperatures. (**b**) Fitted curves of responses to different concentrations of NH_3_ at 280 °C. (**c**) Response to 0.1–100 ppm NH_3_ at 280 °C. (**d**) The response-recovery time to 30 ppm NH_3_ at 280 °C.

The gas sensitivity performance tests of NiFe_2_O_4_, 5 wt% Ag-NiFe_2_O_4_, 10 wt% Ag-NiFe_2_O_4_ and 15 wt% Ag-NiFe_2_O_4_ thin-film gas sensors are shown in Figure 3. The test results of pure NiFe_2_O_4_, 5 wt% Ag-NiFe_2_O_4_, 10 wt% Ag-NiFe_2_O_4_, and 15 wt% Ag-NiFe_2_O_4_ sensors for 50 ppm NH_3_ gas at different temperatures are shown in Figure 3a. Compared to the other sensors, the 10 wt% Ag-NiFe_2_O_4_ sensor exhibits the highest response. Additionally, the response at 280 °C is higher than at other temperatures, indicating that the optimal operating temperature is 280 °C. The fitted curves of the NiFe_2_O_4_, 5 wt% Ag-NiFe_2_O_4_, 10 wt% Ag-NiFe_2_O_4_, and 15 wt% Ag-NiFe_2_O_4_ sensors at 280 °C are shown in Figure 3b. The response curves of the 10 wt% Ag-NiFe_2_O_4_ sensor to NH_3_ (0.1, 0.3, 0.5, 1, 3, 5, 10, 30, 50, and 100 ppm) are shown in Figure 3c, with responses of 1.72, 1.89, 2.02, 2.21, 2.51, 3.06, 3.24, 4.59, 5.36, and 6.14. Figure 3d illustrates the response/recovery times of the NiFe_2_O_4_, 5 wt% Ag-NiFe_2_O_4_, 10 wt% Ag-NiFe_2_O_4_, and 15 wt% Ag-NiFe_2_O_4_ sensors, which are 2 s/19 s, 2 s/12 s, 3 s/9 s, and 6 s/17 s, respectively. The 10 wt% Ag-NiFe_2_O_4_ sensor exhibits exceptional response and recovery characteristics.

**Figure 4:**
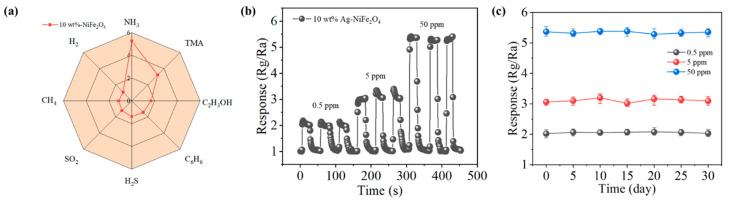
(**a**) Selectivity of 10 wt% Ag-NiFe_2_O_4_ sensor to 50 ppm different gases at 280 °C. (**b**) Repeatability of 10 wt% Ag-NiFe_2_O_4_ sensor at 0.5 ppm, 5 ppm, 50 ppm concentration NH_3_. (**c**) The 30-day long-term stability of the 10 wt% Ag-NiFe_2_O_4_ sensor in response to 0.5 ppm, 5 ppm, 50 ppm of NH_3_.

The selectivity of the 10 wt% Ag-NiFe_2_O_4_ sensor was investigated. As illustrated in Figure 4a, the selectivity curves of the sensor were tested under different gas atmospheres at a concentration of 50 ppm. Compared to the response values for NH_3_ gas, the sensor exhibits lower response values for other gases, indicating good selectivity of the 10 wt% Ag-NiFe_2_O_4_ sensor. This can be attributed to the catalytic effect of the noble metal Ag and the differences in the reactivity of gas molecules at different temperatures. Furthermore, the 10 wt% Ag-NiFe_2_O_4_ sensor demonstrates good repeatability for NH_3_ gas (Figure 4b). Figure 4c presents the long-term stability test curve of the sensor over a period of 30 days. During the testing period, the sensor was placed in a constant temperature and humidity chamber, and a working voltage stress was continuously applied to ensure that the sensor remained in an operational state and a relatively stable experimental environment, during which the sensor demonstrated excellent long-term stability. Table 1 provides a comparison of gas-sensing parameters for various ammonia sensors reported in recent years. This sensor has the advantage of fast response/recovery speeds, which will make ammonia detection quicker and more efficient.

### 3.3. NH_3_ Sensitive Mechanism

The sensitivity performance of Ag-NiFe_2_O_4_ nanocomposites to NH_3_ gas can be explained through the oxygen adsorption model. In this work, the sensor is placed in the air with an operating temperature of 280 °C. The O_2_ molecules in the air will adsorb to form O^−^, as shown in Equations (1)–(3).

O_2_ (gas) → O_2_ (ads) (1)

O_2_ (ads) + e^−^ → O_2_^−^ (ads) (2)

O_2_^−^ (ads) + e^−^ → 2O^−^ (ads) (3)

**Figure 5:**
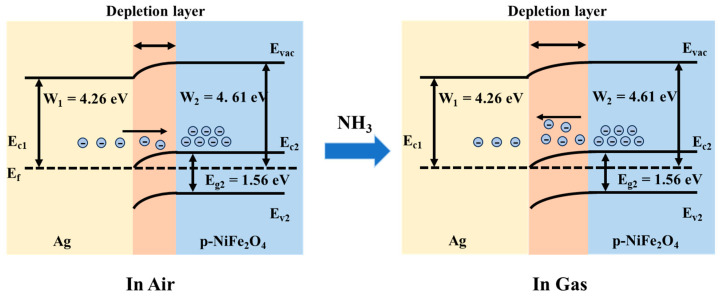
Sensitivity mechanism of Ag-NiFe_2_O_4_ in air and sensitivity mechanism of Ag-NiFe_2_O_4_ in NH_3_ gas.

The resistance change in the Ag-NiFe_2_O_4_ nanocomposite upon NH_3_ adsorption is attributed to the variation in the form of adsorbed oxygen (O^−^) on the material surface. The formation of a Schottky junction between Ag and NiFe_2_O_4_, due to the difference in their work functions, leads to the accumulation of a depletion layer. This results in an increased number of oxygen adsorption sites on the surface of the composite. The catalytic effect of the noble metal Ag also promotes the reaction between NH_3_ and the adsorbed oxygen, thereby enhancing the sensitivity of the composite to a NH_3_. However, as the proportion of Ag increases, the oxygen adsorption sites on the composite surface are occupied by metallic Ag. Excessive Ag content is detrimental to the formation of surface-adsorbed oxygen, thereby weakening the composite’s response to the target gas. Figure 5 shows the mechanism model of NH_3_ gas adsorption on Ag-NiFe_2_O_4_ nanocomposites. Since NiFe_2_O_4_ is a p-type semiconductor, holes are its carriers [27]. When the sensor is in an air atmosphere, free electrons transfer from the lower Fermi level of Ag to the higher Fermi level of NiFe_2_O_4_, forming an electron depletion layer. Ambient oxygen molecules capture electrons from the surface of the composite material to form O^−^. Additionally, due to the oxygen spillover effect of the noble metal, Ag catalyzes the generation of more O^−^. When the sensor is exposed to NH_3_ gas, NH_3_ molecules react with the adsorbed O^−^ on the material surface [28,29]. The response mechanism of the above process is shown in Figure 5. The reaction between ammonia and adsorbed oxygen on the surface of the composite material is shown in Equation 4:

2NH_3_ (ads) + 3O^−^ (ads) → 2N_2_ + 3H_2_O + 3e^−^ (4)

### 3.4. DFT Calculation

**Figure 6:**
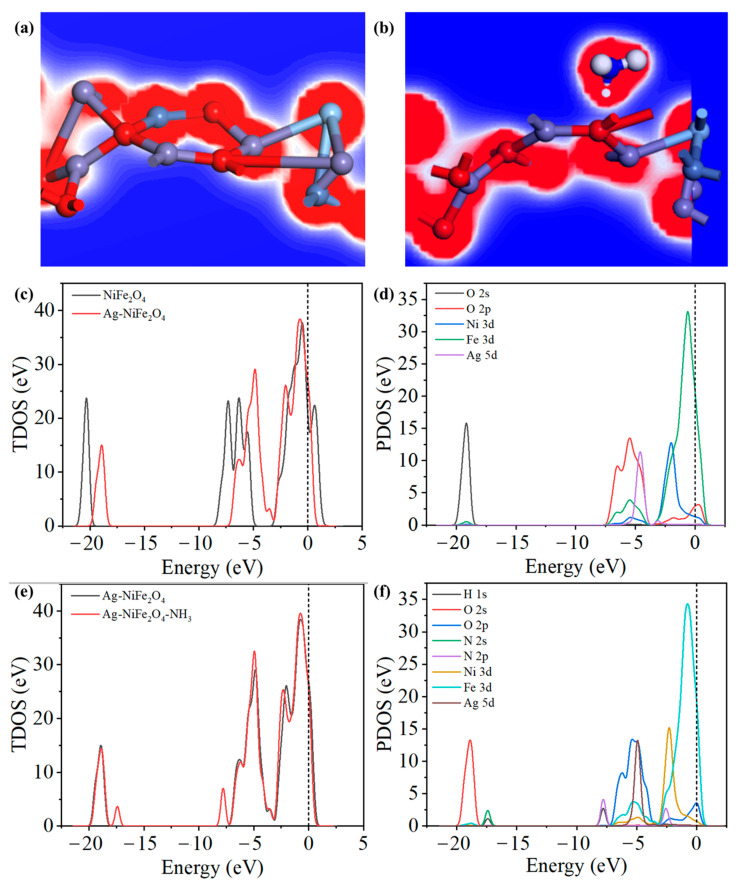
Charge density diagram of (**a**) Ag-NiFe_2_O_4_ and (**b**) Ag-NiFe_2_O_4_ after adsorption of NH_3_. (**c**) The total state density of NiFe_2_O_4_ and Ag-NiFe_2_O_4_. (**d**) Projection state density of Ag-NiFe_2_O_4_. (**e**) The total state density of Ag-NiFe_2_O_4_ before and after NH_3_ adsorption. (**f**) Projected state density after Ag-NiFe_2_O_4_ adsorption of NH_3_.

Based on the XRD characterization results, the crystallographic lattice parameters of the simulation model were determined. First-principle simulations were then performed to calculate the ammonia-adsorption model of the Ag-NiFe_2_O_4_ composite. Structural optimization and energy calculations were carried out for the composite model. Figure 6a,b show the charge density diagrams of Ag-NiFe_2_O_4_ after adsorption of Ag-NiFe_2_O_4_ and NH_3_. The increase and decrease in charge density are represented by the red and blue regions, respectively [30]. As shown in Figure 6c, in comparison to the total density of states (TDOS) of NiFe_2_O_4_, the peaks of Ag-NiFe_2_O_4_ between −20 eV and −3 eV shift to the right, while the peaks between −3 eV and −2.5 eV move slightly to the left. The partial density of states (PDOS) of Ag-NiFe_2_O_4_ is shown in Figure 6d. The rightward shift and weakening of the peak in the range of −20 eV to −17.5 eV is attributed to the O 2s orbital. The peak state density changes significantly within the range of −10 eV to −5 eV, indicating that the electronic state distribution of the material changes after Ag composite, and electron transfer occurs in the composite material. The left shift in the peak within the range of −3 eV to −2.5 eV is affected by Fe 3d and Ag 5d. The TDOS of the Ag-NiFe_2_O_4_ system before and after the adsorption of NH_3_ is shown in Figure 6e,f. After the adsorption of NH_3_ gas, a new peak appears at −17.5 eV, which is generated by the interaction of the H 1s and N 2s orbitals, and a new peak appears at −8 eV, which is generated by the interaction of the H 1s and N 2p orbitals. The peak increase within the range of −7 eV to −4 eV is caused by the N 2p orbitals in NH_3_, and the peak left shift at −2 eV is produced by the combined action of Ni 3d, N 2p, and O 2p orbitals.

## 4. Conclusions

In this study, the 10 wt% Ag-NiFe_2_O_4_ material was synthesized via the hydrothermal method. The Ag-NiFe_2_O_4_ composite was characterized by XRD, SEM, and XPS, and an NH_3_ sensor was fabricated based on this material. At 280 °C, the response of 10 wt% Ag-NiFe_2_O_4_ to 30 ppm NH_3_ was 4.59, with good response and recovery times of 3 s and 9 s, respectively. Among various other gaseous environments, the sensor exhibited the highest response to NH_3_, indicating its superior selectivity. Additionally, the sensor also demonstrates satisfactory repeatability. The sensitivity mechanism towards NH_3_ was further elucidated through first-principles calculations, thereby providing theoretical support for the experimental testing of the Ag-NiFe_2_O_4_ material.

## Figures and Tables

**Table 1:** Comparison of the NH_3_ sensing performances of gas sensors.

Sensing Materials	Temp. (°C)	Conc. (ppm)	Response	Res./Rec. Time (s)	Ref.
WO_3_ porous nanoplates	RT	100	~340	90 s/30 s	[21]
1%Pt/WO_3_	270 °C	100	∼10	7 s/7 s	[22]
β-Ga_2_O_3_	RT	50	219.1%	42.3 s/60 s	[23]
NiCo_2_ZnO_4_	RT	25	22.69	74.84 s/240 s	[24]
α-Fe_2_O_4_/graphene	250 °C	10	13.5%	648 s/152 s	[25]
WO_3_ thin film	250 °C	50	12.58	13 s/34 s	[26]
10 wt% Ag-NiFe_2_O_4_	280 °C	30	4.59	3 s/9 s	This work

## Data Availability

The original contributions presented in the study are included in the article, further inquiries can be directed to the corresponding author.
